# Performance Improvement of a Fiber-Reinforced Polymer Bar for a Reinforced Sea Sand and Seawater Concrete Beam in the Serviceability Limit State

**DOI:** 10.3390/s19030654

**Published:** 2019-02-05

**Authors:** Jiafei Jiang, Jie Luo, Jiangtao Yu, Zhichen Wang

**Affiliations:** College of Civil Engineering, Tongji University, Shanghai 200092, China; jfjiang@tongji.edu.cn (J.J.); luojie1995@tongji.edu.cn (J.L.); wangzhichen@tongji.edu.cn (Z.W.)

**Keywords:** fiber-reinforced polymer (FRP) bar, cementitious composite, concrete cracking, digital image correlation (DIC)

## Abstract

Fiber-reinforced polymer (FRP) has supreme resistance to corrosion and can be designed with optic fibers. FRP can be an alternative to steel reinforcement for concrete structures, and can serve as a sensor for smart concrete structures. Due to poor cracking control and bond performance, the limit of flexural capacity in the serviceability limit state has not been determined, which has obstructed the wider application of FRP bars in smart structures. In this study, in order to overcome these shortcomings, a new engineering cementitious composite (ECC) with superior tensile strain capacity was used to replace the cover around the FRP bars in the tensile zone. To investigate the anti-cracking performance of the new composite beam, seven simply supported beams were designed. In the preliminary investigation, the longitudinal FRP bars in these beams were designed without optic fibers to focus on the mechanical behavior. The beams were tested under four-point load and measured using the digital sensor technique, digital image correlation (DIC). The test results showed that introducing a new ECC layer on the tensile side improves the cracking control and flexural behavior (load capacity and deformability) of a FRP-reinforced sea sand and seawater concrete (SSC) beam, especially in the serviceability limit state. We demonstrate the new composite beam can steadily and fully improve the tensile capacity of FRP bars, which is the basis of using FRP bars as sensors.

## 1. Introduction

Fiber-reinforced polymers (FRPs) possess good mechanical properties, long-term durability, and excellent resistance to corrosion [1,2,3]. Due to the shortage of commercial concrete material supply, sea sand and seawater concrete (SSC) has come to be an alternative to normal concrete (NC). An FRP bar can meet the reinforcement demand for SSC to replace the steel reinforcement. An FRP bar can include optic fibers, which are considered one of the most popular sensing materials. The technique of embedding optic fibers into an FRP bar means the FRP bar itself can serve as a sensor for structural health monitoring as well as the structural reinforcement [4,5,6,7]. It enables intelligent control for construction and monitoring for maintenance, which is especially important for marine structures on islands.

Up to now, limited research on FRP-reinforced SSC structural members have concluded that the behavior of FRP bar-reinforced SSC structural members are similar to those of FRP bar-reinforced NC, as NC and SSC have no significant differences in their macro mechanical behavior. One of major differences between FRP bar-reinforced and steel-reinforced concrete structural members is the service load-to-ultimate load ratio for beams. This ratio in FRP-reinforced concrete beams is much lower than that in steel-reinforced concrete beams [8,9,10,11,12]. Consequently, for FRP bar-reinforced concrete beams, the design is primarily governed by serviceability limit state (SLS) [8,13,14,15,16]. Supposing the applied service load is the same for both steel and FRP-reinforced beams, the beam reinforced by FRP bars needs to be designed with larger dimensions and a larger reinforcement ratio. Researchers found the service load is mostly governed by the maximum crack width [17], compared to the maximum deflection. It reflects the cracking features in FRP bar-reinforced concrete beams is faster increases in crack width. It causes a problem with moisture permeability. As the epoxy in FRP reinforcement is sensitive to moisture, when FRP bar-reinforced structures are exposed to a marine environment, it still brings durability issues for FRP bars. This cracking pattern is determined by the bond performance between FRP bars and concrete, which is comparatively poorer than that between steel bars and concrete [18,19,20]. The resulting larger crack widths also induces high strain concentration in the FRP bar near the crack mouth. Therefore, FRP reinforcement fails at a much lower strain than the rupture strain, which is determined using a direct tensile test. This phenomenon impairs the efficiency of reinforcement and the service behavior of embedded optical fibers under service load. To summarize, the poor bonding performance and control of cracking poses barriers for the use of FRP bars as reinforcements and sensors for smart structures.

In the past decade, efforts have been taken to improve cracking control in the reinforced beams. Except the prestressing technique, introducing high performance cementitious materials was proposed and investigated in some studies. In particular, engineered cementitious composites (ECC) were investigated for controlling the flexural crack width in steel reinforced concrete beams [21,22,23,24,25,26,27,28,29,30]. Moreno et al. [29] found the interaction between the rebar and ECC reduces the fracture strain of the ECC and bar. Therefore, a larger fracture strain is required for ECC in bar-reinforced ECC than ECC itself under direction tension. Yuan et al. [30] reported a pioneering investigation on the effect of ECC reinforcement on FRP bar-reinforced concrete beams, but focused on the ultimate performance. In 2016, a new category of ECC, named as ultra-high ductile cementitious composites (UHDCC), was developed by Yu et al. [31]. The tensile strain capacity of UHDCC ranges from 8% to 12%, and the uniaxial tensile strength ranges from 4 MPa to 20 MPa [31,32,33,34]. The pioneering study by Yu et al. showed the flexural performance of a UHDCC beam can behave like an ordinary reinforced concrete beam, with a reinforcement ratio of 1.51% and small cracking width [35,36]. The experimental studies above demonstrated the feasibility of using ECC for crack width control. 

Considering the points above, a novel composite system, an FRP bar-reinforced SSC beam, is proposed in this study. A UHDCC layer was adopted to replace the concrete in the tensile zone and act as the matrix for the FRP bar. As the optic fibers do not affect the tensile behavior of FRP bars, they were not designed for the FRP bars in the mechanical investigation for simplicity. The crack control capacity of UHDCC was expected to improve the performance of the composite beam, especially in the serviceability limit state. To confirm the properties of the new composite beam, four-point bending tests were conducted on simply supported beams. Beams with and without UHDCC were designed to investigate the effect of the UHDCC layer on the flexural behavior in terms of characteristic load capacity, crack width, deformability, etc.

## 2. Material Fabrication and Properties

### 2.1. Concrete

In this study, an SSC was designed for the specimens. The mixture included cement, coarse aggregate, sea sand, and seawater. The coarse aggregate was well-graded crushed granite, as used in NC. The sea sand was brought from Ningbo costal area in the east of China, and the seawater was artificial water, which was a mixture of dissolved sea salts to meet the target salinity of 3%. The mix design was created referring to the “Specification for mix proportion design of ordinary concrete (JGJ55-2011)” [37] for a target strength of 30 MPa (150 mm cube strength). Experiments were conducted in a standard laboratory. The laboratory mix proportion was adjusted with a high-range water-reducer (HRWR, also called a super plasticizer) to meet the slump requirement for casting on-site (Table 1). SSC cubes (150 mm) were cast at the same time as the SSC beams, and all were cured outdoors together. The compressive strength was tested on the same day as beam loading, which was 70 days after the casting. The average strength was 30.49 MPa with a standard deviation of 2.59 MPa. The macro mechanical behavior showed minor differences compared to NC. The micro level difference between SSC and NC and its effect on the long-term behavior of FRP-reinforced beams are not within the scope of the study.

### 2.2. Ultra-High Ductile Cementitious Composites 

**Figure 1:**
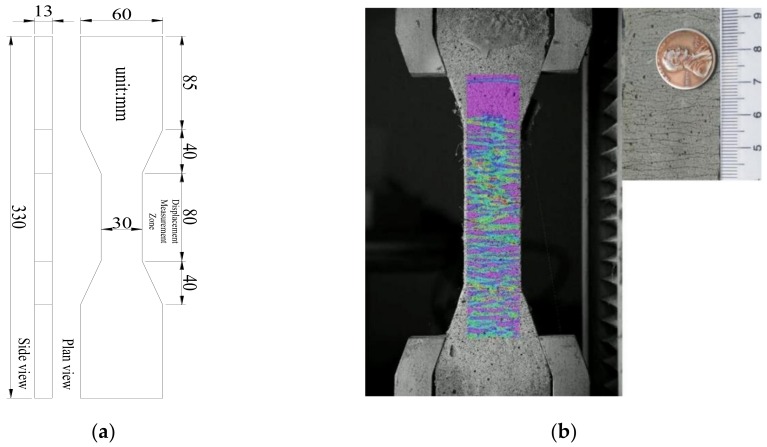
(**a**) Geometric size and (**b**) crack pattern of UHDCC dog bone specimen.

**Figure 2:**
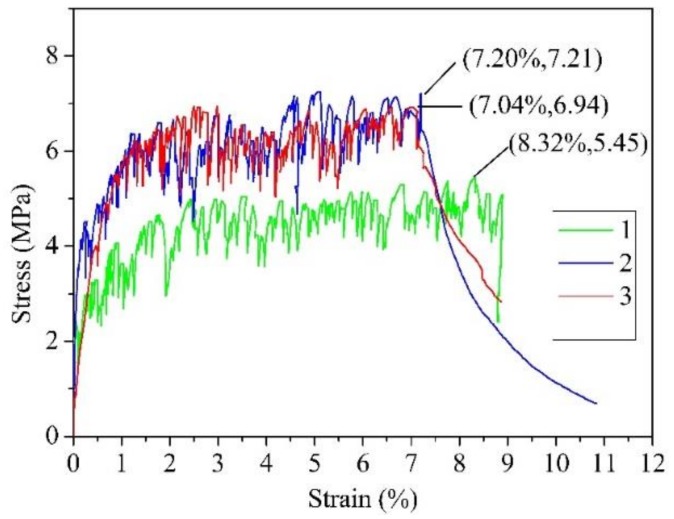
Typical stress–strain curve of the UHDCC dog bone specimen under tension.

The mixture of UHDCC included PO 52.5 cement, class II fly ash, fine sand, water, polyethylene (PE) fibers, and HRWR. In order to maintain the chlorine content in UHDCC close to that in SSC, the sea sand and seawater from SSC were used to replace the original ingredients. Sea sand was screened to the required fineness for UHDCC. The sands had the largest size smaller than 0.21 mm. The mix proportion of UHDCC is presented in Table 2. PE fibers with high strength and high Young’ modulus were used to reinforce cementitious matrix (Table 3). The volume fraction of the PE fiber was 2%. Five dog-bone shaped specimens were cast according to recommendation from the Japan Society of Civil Engineers (JSCE) [38]. All the specimens were cured outdoors for 70 days and loaded to failure under increasing tension (Figure 1). Dog bone specimens exhibit strain-hardening characteristics (typical stress-strain curves in Figure 2) with multiple cracks. Since the UHDCC was mixed manually, the uniformity of the mixture could not be guaranteed, which caused the cracking behavior of specimen 1 to occur earlier than in specimens 2 and 3. The test results indicated that the cracking strength of UHDCC was 3.03 MPa with a corresponding strain of 0.20%. The cracking strength was close to that of SSC concrete. The peak tensile strength reached 6.25 MPa. The tensile strength was three to five times that of the conventional concrete. More importantly, the fracture strain was 7.79%, which far exceeds the elongation of the FRP bar. It is noted that the average crack width at the peak strength was less than 0.2 mm, creating a highly durable protection for longitudinal reinforcement in a wide variety of environmental exposure conditions. 

### 2.3. Basalt Fiber–Reinforecd Polymer (BFRP) Bars

**Figure 3:**
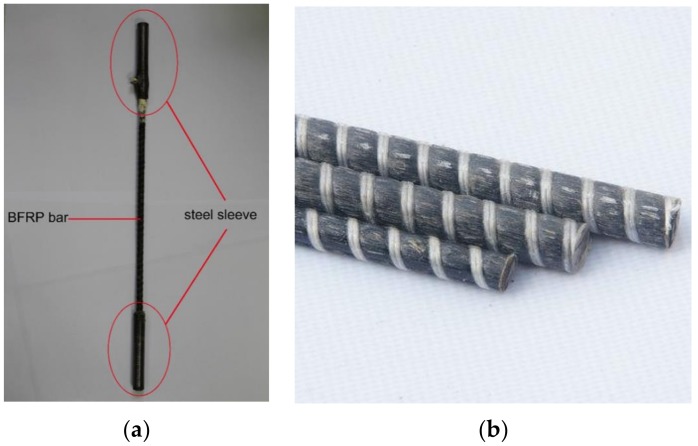
Basalt fiber–reinforced polymer (BFRP) bars: (**a**) BFRP specimen in tensile test; and (**b**) BFRP bars with different diameters.

Ribbed BFRP bars with three different diameters were adopted in this study, as shown in Table 4. They were manufactured without optic fibers by Jiangsu Green Materials Vally New Material T&D Co., Ltd., China. The BFRP bars were basalt fibers bonded with vinyl resins. To determine the tensile behavior of the BFRP bar, steel sleeve grouting was used to reinforce both ends of the BFRP bar for clamping (Figure 3). The anchorage length of the steel sleeve followed the “Glass fiber reinforced plastics rebar for civil engineering (JG/T 406-2013)” guideline [39] to avoid pull-out failure. To enhance the friction between the BFRP bar and steel sleeves, the surface of the sleeve was cut thread. For each type (diameter) of BFRP bar, five identical specimens were prepared and tested using the tensile testing machine. Table 4 lists the ultimate tensile strength, ultimate strain, and modulus of elasticity. The elongation of BFRP bars was lower than that of UHDCC.

## 3. Design and Preparation of Beam Specimens

**Figure 4:**
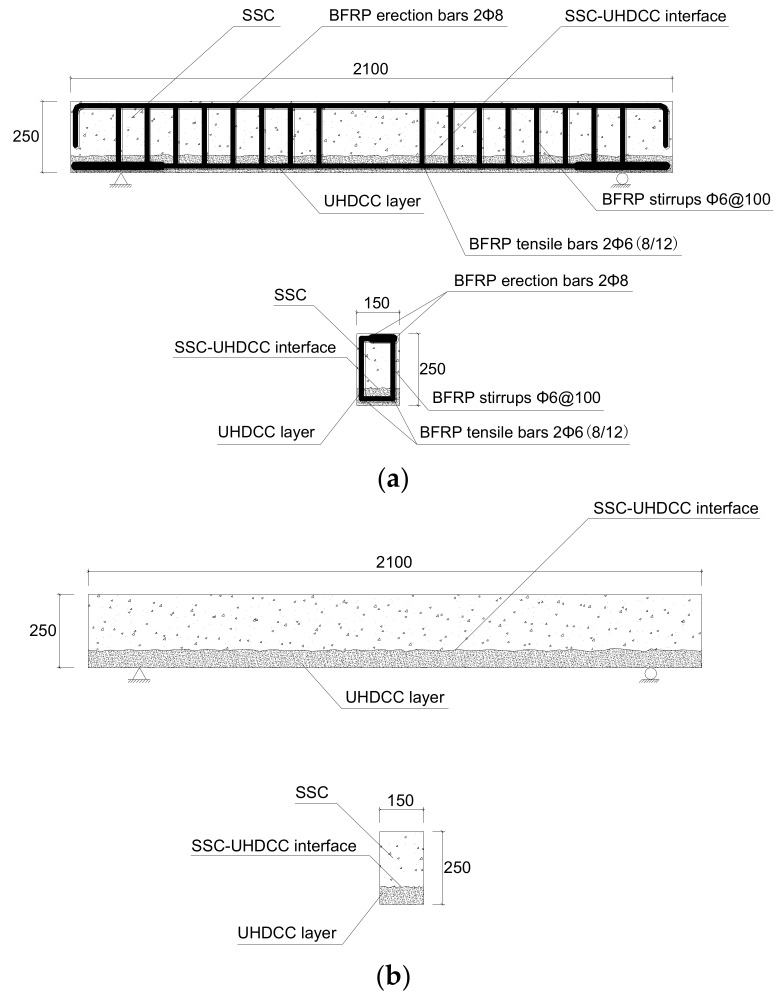
Details of sea sand ans seawater concrete (SSC)-ultra-high ductile cementitious composites (UHDCC) specimen (unit, mm). (**a**) SSC-UHDCC beams with BFRP reinforcement; and (**b**) SSC-UHDCC beams without BFRP reinforcement.

Seven specimens were designed for the four-point bending test to investigate the flexural performance of beams with different configurations. The dimensions of beams were uniform (i.e., b × h × L = 150 × 250 × 2100) (Figure 4). All the longitudinal bars and stirrups were BFRP bars. Three SSC specimens were reinforced using BFRP bars with the diameters of 6, 8, and 12 mm, respectively. The other four SSC-UHDCC specimens were designed to have 60-mm-thick UHDCC layers at the bottom side instead of the original SSC part. Among them, the SSC-UHDCC-plain beam had no longitudinal bar inside the UHDCC layer (tensile region). It was designed to check the flexural reinforcement contribution from UHDCC itself. All the beams had the BFRP stirrup, 6 mm in diameter and 100 mm in spacing, in the shear-moment zone. It was designed according to the Chinese code “Technical code for infrastructure application of FRP composites (GB 50608-2010)” [40] to avoid shear failure. The details of specimens are presented in Table 5. 

**Figure 5:**
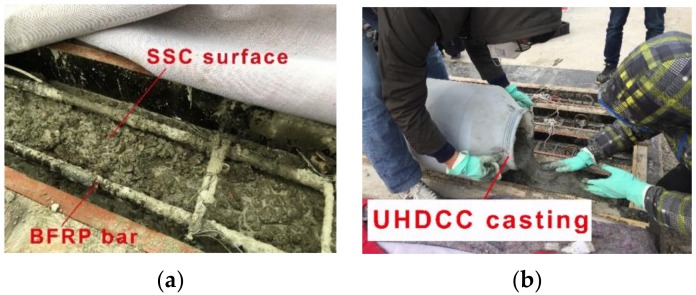
Casting of SCC-UHDCC beam: (**a**) casting of SSC layer; and (**b**) casting of UHDCC layer.

Beam specimens were cast in the laboratory of Tongji University, (Shanghai, China). The reinforcement cage was fixed in the formwork with the tensile bars facing upward. Then, the SSC was cast for all seven beams. For SSC-UHDCC beams, the SSC was cast initially and finished at the height of the interface between the SSC and UHDCC. In order to produce a good interface bond between SSC and UHDCC, the SSC surface was artificially roughened to partially expose coarse aggregates (Figure 5). The specimens with SSC sections were first cured for one week, and then UHDCC layers were cast for four SSC-UHDCC specimens. After the casting, all the specimens were cured outdoors.

## 4. Beam Testing

### 4.1. Test Program

**Figure 6:**
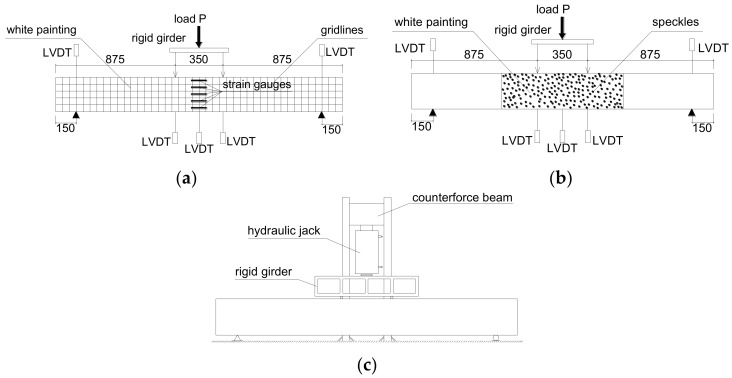
Beam test set-up and instrumentation (unit, mm): (**a**) lateral side A; (**b**) lateral side B; and (**c**) loading system.

Four-point loading tests were conducted on all the beams after a 70-days curing period. A hydraulic jack was used to apply a concentrated load that was divided into two by a rigid girder with a span of 350 mm. The span between two supports was 1800 mm. The setup of instrumentation is illustrated in Figure 6. A load cell was attached to the hydraulic jack to record the load values during testing. The deflection of beam was recorded by five linear variable differential transformers (LVDTs) installed in the pure bending zone or at supports. For each beam, five strain gauges (50 mm gauge length) were pasted onto the lateral surface (side A in Figure 6a) of cross section at mid-span, and two strain gauges were attached to the surface of each BFRP tensile bar. The digital image correlation (DIC) method was used as the sensor to record the full field deformation and the crack development process during testing. DIC was shown to be a reliable method for reinforced concrete specimens in recent research [41,42,43,44,45]. Before DIC sensing, the pure bending moment (PBM) zone on the other side, (i.e., side B in Figure 6b), was set as the area of interest, which was sprayed with white paint and random black speckles. A digital single lens reflex camera recorded raw images every five seconds until the end of testing.

### 4.2. Loading Process

According to “Standard for test method of concrete structures (GB/T 50152-2012)” [46], the displacement-control loading program was used in this test (Figure 6c). The load interval was set to 1.8 kN for the experiments on the seven beams. During each step of loading, the cracks locations and development were marked on side A.

## 5. Test Observation

Cracking development was described based on the observation on the side A surface. Typical values, such as the cracking and peak load, were recorded from the load cell. The failure mode was determined by the observation of failure process. 

### 5.1. SSC Beams

**Figure 7:**
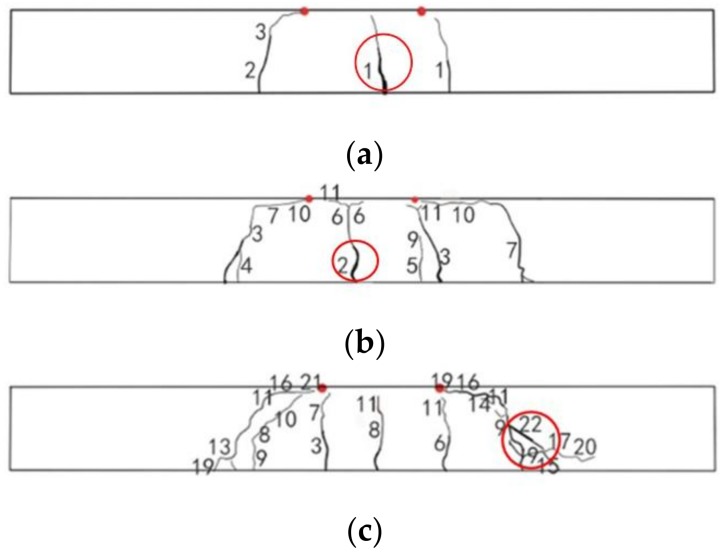
Crack distribution on side A surface of SSC beams (the two red points are loading points): (**a**) SSC-6; (**b**) SSC-8; and (**c**) SSC-12.

SSC-6 was designed to be an under-reinforced beam with a reinforcement ratio of 0.17%. The theoretical upper limit for under-reinforcement is 0.207%. One flexural crack appeared in the middle of beam and developed quickly to around half of the height of the beam section. Shortly after, another two cracks appeared near the PBM zone. When the middle crack stretched near the top of the beam section, one of the tensile BFRPs ruptured with a loud sound and the test was terminated. The peak load was 11.3 kN. The largest crack width originated from the middle flexural crack (red circle in Figure 7a). The concrete in the compressive zone remained intact. The average rupture strain was at 1.28%, which is only 50.79% of the strain of BFRP bars under direct tensile testing. The distribution of cracks is presented in Figure 7a.

**Figure 8:**
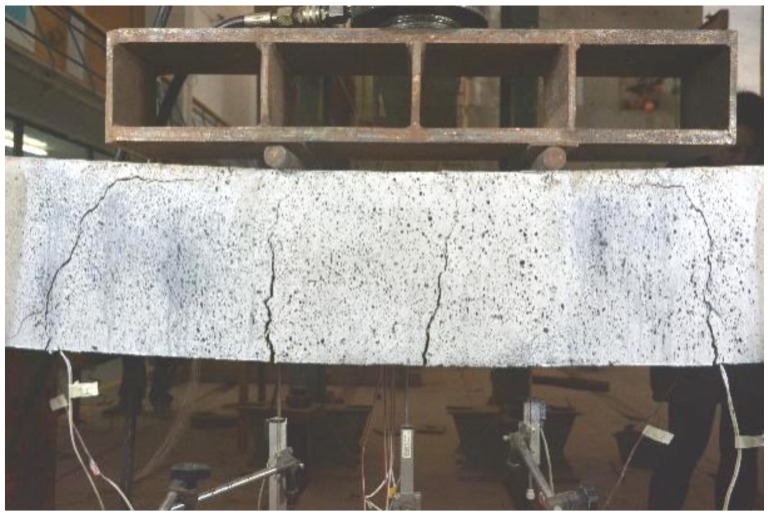
Failure of SSC beams.

The reinforcement ratio of SSC-8 was 0.3%, which was higher than the critical reinforcement ratio of under-reinforcement 0.223%, but lower than 1.5 times of the ratio (that is 1.5 × 0.233% = 0.334%). The failure of beam may be caused by either the rupture of tensile bars or the crush of the concrete [47]. The first flexural crack appeared in the PBM zone at a load of 3.53 kN. When the load increased to around 4.8 kN and 9 kN, another two flexural cracks appeared in the PBM zone, and the first crack stretched to over half the height of beam. One diagonal crack developed from the middle height of the beam. When the load reached 12.6 kN, the diagonal crack suddenly changed its direction horizontally toward the loading point. Simultaneously, another flexural crack was generated in another shear moment (SM) zone and changed its direction as the shear crack when the load was around 18 kN. The height of the compressive zone decreased dramatically and one of the tensile BFRP bars ruptured with a loud sound, and the beam failed. The average rupture strain was at 1.51%, which is 58.30% of the strain of BFRP bars under direct tensile testing. The crack with the largest width was the flexural crack in the middle (red circle in Figure 7b). The peak load was 27.58 kN. Accordingly, the failure mode is the flexural failure of under-reinforced beam. The distribution of cracks is presented in Figure 7b and the image at failure is presented in Figure 8.

SSC-12 was designed to be an over-reinforced beam with a reinforcement ratio of 0.69%. The first crack was a flexural crack at the edge of PBM zone. The cracking load was 3.83 kN, and the second and third cracks appeared in the PBM zone at loads of around 10.8 kN and 14.4 kN, respectively. Following that, two diagonal cracks appeared in the SM zone. These cracks initiated in the middle height of beam section. Finally, one diagonal crack with the largest crack width developed at the loading point. Then local crush at the loading point occurred. The peak load was 47.05 kN. The failure of the beam was caused by insufficient shear capacity, which was not expected. This could have been caused by overestimation of bent stirrups capacity. The reason for this phenomenon was outlined by ISIS Canada (2001) [11]. The tensile strength at the bend was dramatically weaker due to stress concentration. However, the reduction effect was not well predicted by the code. The crack with the largest width is denoted by a red circle in Figure 7c. The distribution of cracks and image at failure are presented in Figure 7c.

### 5.2. UHDCC-SSC Composite Beams

**Figure 9:**
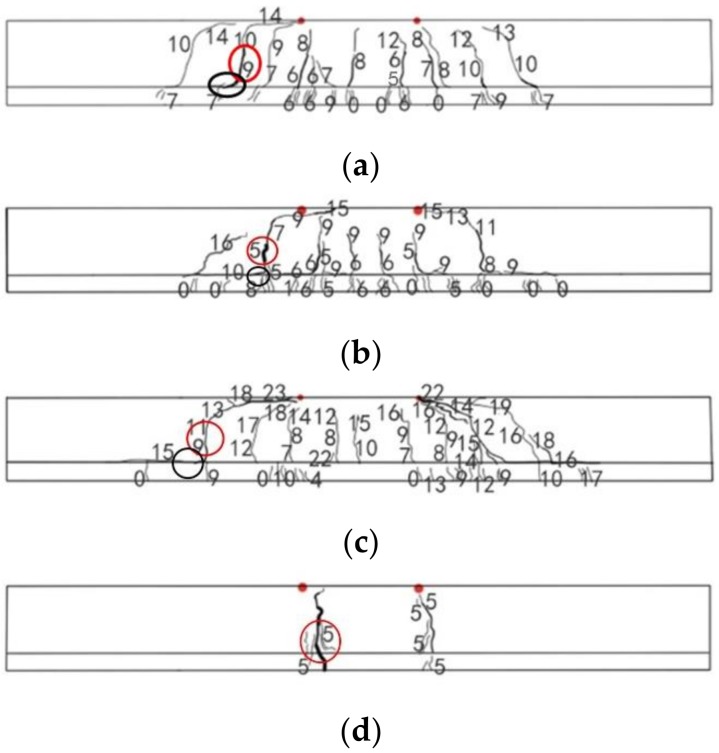
Crack distribution on side A surface of SSC-UHDCC beams (the two red points are loading points): (**a**) SSC-UHDCC-6; (**b**) SSC-UHDCC-8; (**c**) SSC-UHDCC-12; and (**d**) SSC-UHDCC.

**Figure 10:**
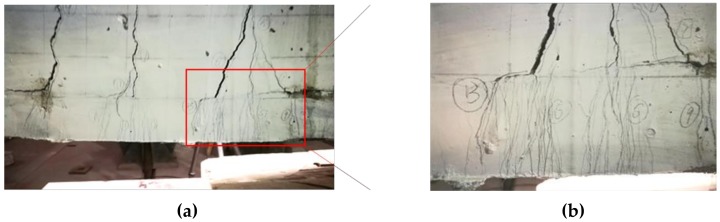
Failure of SSC-UHDCC beams (side B surface): (**a**) SSC-UHDCC-6; and (**b**) detail.

Comparatively, all of the SSC-UHDCC beams reinforced with BFRP bars initially displayed micro cracks densely distributed in the middle of UHDCC layer. Then, several micro cracks merged into one macro crack at the interface, which further stretched toward the compressive zone of the beam (Figure 9a–c). The cracking load was recoded when the first macro crack initiated at the interface. The typical crack distribution in SSC and UHDCC are presented in Figure 10a,b, respectively.

During the test of SSC-UHDCC-6, flexural cracks initiated early as the load reached 9.0 kN, which was considered the cracking load in this study. Afterward, several flexural-shear cracks were generated in the SM zone at a load of 16.2 kN. It is of interest to note that the macro cracks that occurred in the SSC part were smeared in numerous micro cracks in the UHDCC layer within both the PBM and SM zones. Several groups of micro cracks progressively merged into 10 macro cracks from the interface (Figure 9a). Detachment at the interface was observed in the original tip of one shear-flexural crack but without obvious horizontal slip (black circle in Figure 9a). Then, the flexural cracks progressively developed to 4/5 of height of beam and ceased to develop until the load of 21.6 kN. Afterward, shear-flexural cracks continued to extend toward the adjacent loading point until failure. Finally, the concrete around one loading point crushed. The peak load was 32.43 kN. The final crack distribution shows the widest crack was one flexural-shear crack (red circle in Figure 9a). In comparison with SSC-6, SSC-UHDCC-6 had a larger cracking load, more cracks, but much smaller crack spacing. Above all, the change in failure mode from flexural failure to shear failure is due to the enhanced flexural capacity higher than the shear capacity. 

In SSC-UHDCC-8, groups of micro cracks progressively merged into eight macro cracks at the edge of interface (Figure 9b). Both flexural and shear-flexural cracks were observed early when the load reached the cracking load of 9.0 kN. Comparatively, more flexural cracks developed afterward. These cracks extended to 2/3 of height of beam and ceased to develop when the load was around 16.2 kN. Afterward, the extension of two flexural-shear cracks dominated the cracking development. The beam failed when the two cracks reached the adjacent loading points, accompanied by local concrete crushing at the loading point. The peak load was 39.55 kN. Detachment at the interface was also observed at the tip of two shear-flexural cracks with the largest opening width of 2.07 mm (black circle in Figure 9b). The widest crack is indicated by the red circle in Figure 9b. Similar to SSC-UHDCC-6, the failure mode of SSC-UHDCC-8 was shear failure, and fewer macro cracks occurred than in SSC-UHDCC-6. In comparison with SSC-8, the composite beam had a larger cracking load and more cracks, but smaller crack spacing. 

In SSC-UHDCC-12, a similar micro cracking pattern was observed during the early loading process. The micro cracking region in the UHDCC layer was longer than in the other two composite beams. Groups of micro cracks progressively merged into 9 macro cracks from the interface (Figure 9c). Two flexural cracks appeared first at the load of 12.6 kN. Later, three flexural-shear cracks generated at a load of around 14.4 kN. At that time, detachment at the interface was also observed. The largest opening was around 2.92 mm (black circle in Figure 9c). After the major flexural crack stopped at 4/5 the height of the beam at the load of 21.6 kN, the flexural-shear cracks began to decline toward the adjacent loading points. Finally, the widest crack was one flexural-shear crack (red circle in Figure 9c). The beam failed with the observation of local concrete crushing at the loading point. In this beam, the peak load was 49.67 kN. Similar to SSC-12 and other FRP-reinforced SSC-UHDCC beams, the failure mode was shear failure. In comparison to SSC-12, SSC-UHDCC-12 had a larger cracking load and more cracks but smaller crack spacing. 

In SSC-UHDCC-plain, dense micro cracks first appeared in the UHDCC layer. Then, only two flexural cracks generated in the PBM zone. The cracking load was 9.0 kN. Following that, two cracks quickly extended from the interface to the compressive zone of the beam, which caused concrete in tensile section to immediately lose tensile bearing capacity. Finally, the strength capacity of the UHDCC cover was not high enough to balance the tensile demand transferred from the cracked concrete, and the beam immediately lost load bearing capacity as an under-reinforced beam. The peak load was 12.49 kN, which was close to the load capacity of SSC-6. The widest crack is indicated by a red circle in Figure 9d. However, the beam did not split into two segments, which illustrates that the UHDCC layer with a higher fracture strain bonded well with the SSC.

From the tests, the development process of the cracks and critical load values were observed and recorded. From the cracking features of BFRP bar-reinforced composite beams, we found that in SSC-UHDCC beams, the number of cracks increased and the average crack spacing decreased. More energy was consumed during the loading of the SSC-UHDCC beam compared with the counterpart SSC beam (Figure 10). Hence, the load and deformation capacity were enhanced due to the UHDCC layer. The load capacity of SSC-UHDCC-plain was close to that of SSC-6, demonstrating that the UHDCC layer can function as tensile reinforcement. The under-reinforcement failure mode of SSC-6 and SSC-8 was expected. The failure mode of SSC-12 and all the SSC-UHDCC beams were shear failure, as the shear capacity was overestimated and lower than the improved flexural capacity. In general, the UHDCC layer can enhance the flexural capacity of a beam reinforced with BFRP bars. 

## 6. Discussion

**Figure 11:**
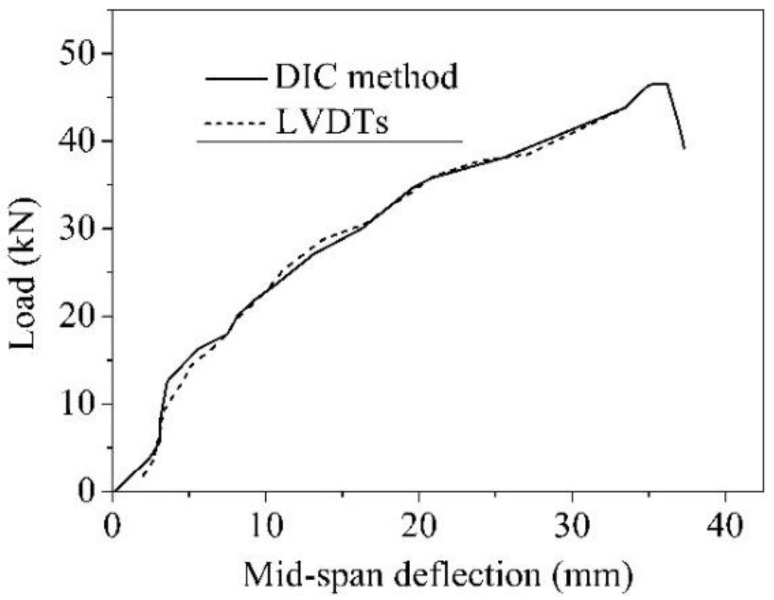
Comparison of deflection from digital image correlation (DIC) method and linear variable differential transformers (LVDTs) (SSC-12).

LVDTs and DIC technology (see Figure 6) were simultaneously used to acquire the deformation responses of beams under loading. As shown in Figure 11, the displacement data obtained from the DIC agree well with those from LVDTs, demonstrating the reliability of DIC in reflecting the full-field deformation. Hence, the deflection is based on the DIC results. 

**Figure 12:**
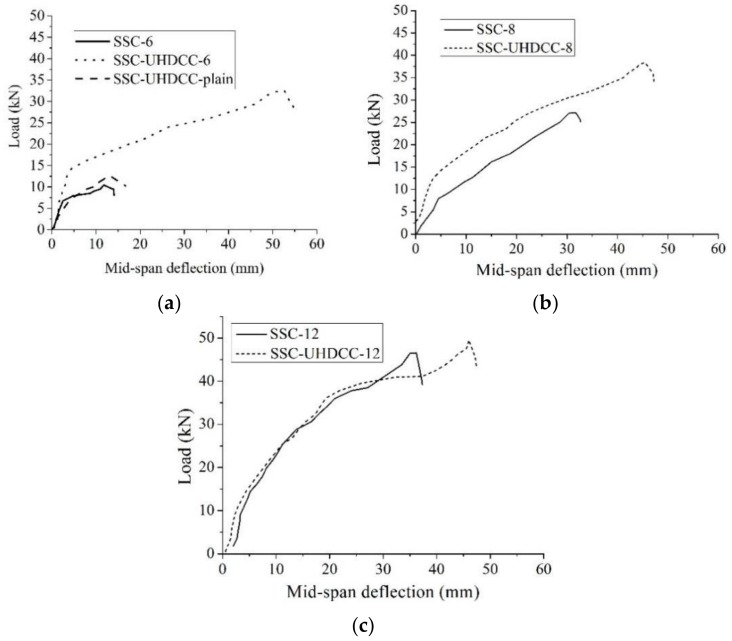
Load vs. displacement curve: (**a**) SSC-6 vs. SSC-UHDCC-6 vs. SSC-UHDCC-plain; (**b**) SSC-8 vs. SSC-UHDCC-; and (**c**) SSC-12 vs. SSC-UHDCC-12.

Figure 12 shows the load-deflection curves of all tested beams. Based on the test data, analyses were conducted to explore the effect of UHDCC layer on the mechanical properties of SSC beams.

### 6.1. Cracking Load Capacity 

**Figure 13:**
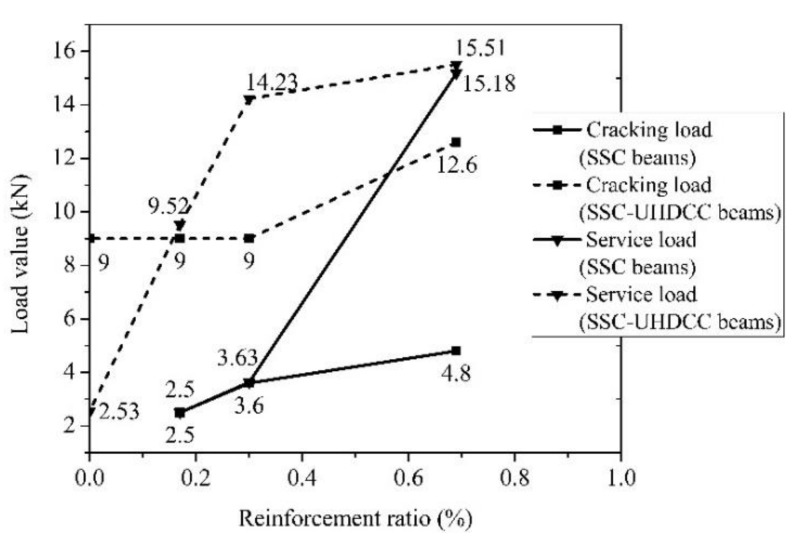
Cracking load and service load of beams.

All the cracking loads were based on the cracking observation on the side A surface and inferred from the recorded load step. The cracking loads of SSC-6, SSC-8, and SSC-12 were 2.5 kN, 3.60 kN, and 4.80 kN, respectively. The cracking loads of UHDCC-SSC-6, UHDCC-SSC-8, UHDCC-SSC-12, and UHDCC-SSC were 9.0 kN, 9.0 kN, 12.6 kN, and 9.0 kN, respectively. For two kinds of beams, the cracking load increased with the increase of reinforcement ratio. The cracking loads of UHDCC-SSC beams were much higher than those of SSC beams (Figure 13). The enhancement ratio due to the UHDCC layer ranged from 150% to 260%, and slightly decreased with the increase of reinforcement ratio. Hence, the first advantage of introducing the UHDCC layer is the increase in the cracking load.

### 6.2. Service Load Capacity 

For FRP-reinforced concrete beams, the SLS usually governs the final design. Many studies focused on how to define service load. To summarize, there are four criteria: (1) mid-span deflection [16], (2) service strain in FRP bars [17], (3) the maximum crack width [15,48], and (4) peak load (*P_m_*) divided by the load factor 1.5 [49]. In the second criterion, the service strain in FRP bars is determined by the maximum cracking width. The value of 0.002 is recommended for FRP bars [17], which corresponds to the maximum crack width of 0.5 mm. In the third criterion, the maximum crack width is controlled to ensure adequate structural performance and sufficient durability of the structure [50]. The maximum crack width for beams with FRP bars is larger than for steel reinforcement, as FRP material has good anti-corrosion performance. ACI 440.1R-06 regulates the maximum crack width as 0.5 mm for interior exposures and 0.7 mm for exterior exposures [15], whereas JSCE recommends a maximum crack width of 0.50 mm for both interior and exterior exposure [48]. In this study, the threshold value for the maximum width was taken as 0.5 mm. Hence, the second and third criteria are fundamentally the same. All typical values from four criteria are summarized in Table 6. Firstly, the service load showed differences between the service strain and crack width criteria, demonstrating that a fixed service strain is not always consistent with the same crack width control. The service strain is influenced by the bond characteristics of bars, bar spacing, and bar size [17]. This phenomenon was also observed by El-Nemr et al. [51]. Accordingly, the value calculated by the crack width criterion was taken as the characteristic value for the two criteria for all specimens. It can be seen that the most conservative value for SLS was obtained from either the deflection or crack width criterion. For the demand at floor level, the service load for shear failure dominated beams (SSC-12, UHDCC-SSC-8, and UHDCC-SSC-12) is governed by the lower bound of deflection, whereas the others are governed by the crack width. For the beam at the roof level, the deflection limit is *L*/180 (*L* is the span) [16,52]. Then, all the beams’ SLS capacities were governed by the maximum crack width, except for UHDCC-SSC-12. 

In general, the service load increases with the growth in the reinforcement ratio for both SSC beams and UHDC-SSC beams. At a fixed reinforcement ratio, the UHDCC layer efficiently contributes to the service load, as expected. The enhancement ratios of service load in UHDCC-SSC-6, UHDCC-SSC-8, and UHDCC-SSC-12 were 281% for roof level, 292% (332% for roof level), and 111% (36.7% for roof level) compared to the counterpart SSC beams, respectively. The percentage of service load to peak load also increased. Although the ratio is still below the ratio in reinforced concrete (RC) structures, the service load is enhanced without changing the beam dimensions or the FRP reinforcement ratio. This exhibits another main benefit of the UHDCC layer.

### 6.3. Ultimate Load Capacity and Corresponding Deflection in Under-Reinforcement Failure Mode

**Figure 14:**
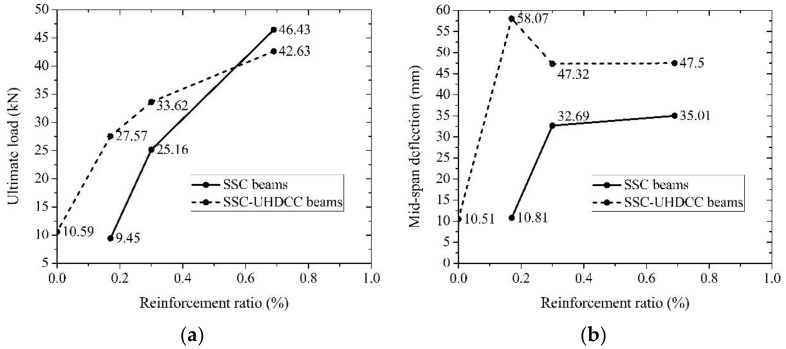
Ultimate load and mid-span deflection of beams: (**a**) ultimate load; and (**b**) mid-span deflection.

Figure 14a illustrates the influence of UHDCC and the reinforcement ratio on the ultimate load bearing capacity of beams. In Figure 14a, the SSC-UHDCC-plain beam provides as much load capacity as SSC-6. In other words, due to the high tensile strain capacity and tensile stress of UHDCC, the 60 mm plain, UHDCC layer contributes a longitudinal BFRP bar of the reinforcement ratio as 0.17%. Therefore, compared with SSC-6 and SSC-8 beams, SSC-UHDCC-6 and SSC-UHDCC-8 increased their ultimate load capacity by 192% kN and 34% kN, respectively, as shown in Table 7. Unfortunately, both SSC-12 and SSC-UHDCC-12 suffered shear failure instead of flexural failure. The premature shear failure led to insufficient use of the UHDCC and BFRP bar. Consequently, a minor difference was found in the shape of the load-deflection curve. The ultimate load capacity only decreased by 8%. 

All the SSC-UHDCC beams experienced larger deflection than SSC beams at ultimate load (see Figure 14b). The UHDCC layer had a much more significant effect on the deformability of beam than on the ultimate load capacity. The enhancement ratios of the deflection at ultimate load for SSC-UHDCC-6, 8, and 12 beams were 437%, 45%, and 36%, respectively. Similar to the situation in load capacity, tremendous improvement in deformability was achieved for the less-reinforced beam (SSC-6 vs. SSC-UHDCC-6). 

In summary, three characteristic loads and deformation are summarized in Table 7 for evaluating the effect of UHDCC layer. In general, the UHDCC layer not only serves as additional FRP bars in force strengthening, but also further improves the deformability of the SSC beams with the same equivalent reinforcement ratio.

Beams incorporated with UHDCC layers exhibited much better deformability. This brings up a question: How can the UHDCC layer enhance the deformability of a composite beam without changing the rupture elongation of the BFRP bar? To answer this, further analysis is conducted on the crack pattern of SCC and UHDCC matrix and the strain distribution in BFRP and UHDCC.

### 6.4. Crack Pattern 

A more detailed picture of cracking development in the SSC part of all the beams except SSC-6 was captured using the DIC method. The crack width was calculated by the method proposed by Hu and Wu [53]. The crack in SSC-6 emerged once the load was added and the crack width developed so quickly that the camera failed to capture the precise strain field using the DIC method, so only the biggest crack width of SSC-6 was obtained.

**Figure 15:**
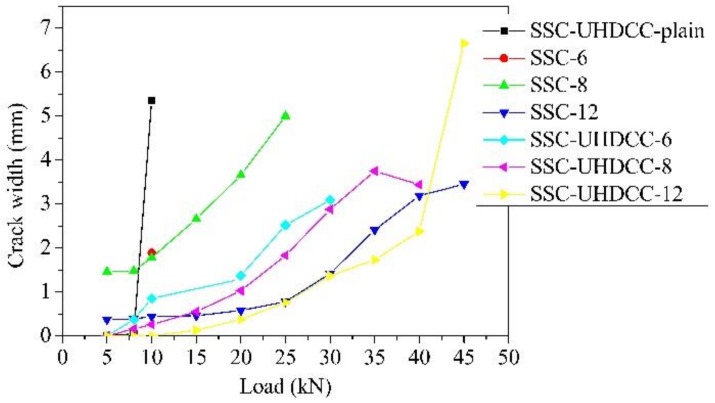
Crack width under different load levels.

Figure 15 illustrates the development of crack width in the SSC part during loading. The specific crack width and crack number in the SSC part are presented in Table 8. The number of cracks in the SCC part of the SSC-UHDCC beams significantly increased compared to the SSC beams, thus leading to fine cracks. The crack numbers in the UHDCC layer were at least one order of magnitude more than those in the adjacent SSC parts. As shown in Figure 10, one crack in SSC dispersed into more than 10 fine cracks in UHDCC. This indicates that the crack width in UHDCC is far smaller than in the adjacent SSC part, which was already smaller than that in SSC beams.

In both SSC beams and SSC-UHDCC beams, the flexural crack width decreased with the increase in reinforcement ratio. When the SSC-UHDCC-plain beam was in the ultimate state, although there were 54 cracks in the UHDCC layer, there were only two cracks in the SSC part, close to that of SSC-6. This implies that the UHDCC layer without any longitudinal reinforcement does not trigger multiple cracks in the SSC part. 

In general, the combination of UHDCC and FRP has enormous advantages in crack width control compared with beams solely reinforced with FRP bar or UHDCC. Crack number and width are highly related to the magnitude of the combined reinforcement ratio, which agrees with logical crack width control.

**Figure 16:**
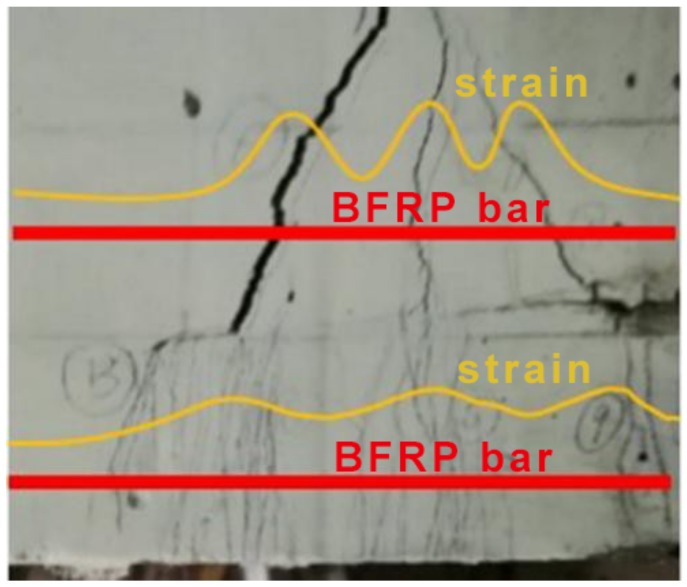
Strain distribution assuming two BFRP bars (red solid line) are embedded in the SSC and UHDCC layer.

Small crack width and crack spacing can alleviate strain (also stress) fluctuation along longitudinal reinforcement. A sketch is presented in Figure 16 to illustrate the difference assuming two BFRP bars (red solid line) are embedded in the SSC and UHDCC layer in the PBM zone. In NC, the strain of the reinforcement near the crack is obviously larger than between adjacent cracks, since all the tension contribution is lost at the crack mouth. In UHDCC, the crack width and the crack spacing are much smaller, and the tension contribution of UHDCC still exists at the crack mouth; therefore, the strain fluctuation is smoothed. This explains why the SSC-UHDCC combination can effectively enhance the deformability of a beam. To quantitatively verify this point, we completed an analysis based on experimentally obtained strain fluctuation in the following section.

### 6.5. Strain in the PBM Zone

**Figure 17:**
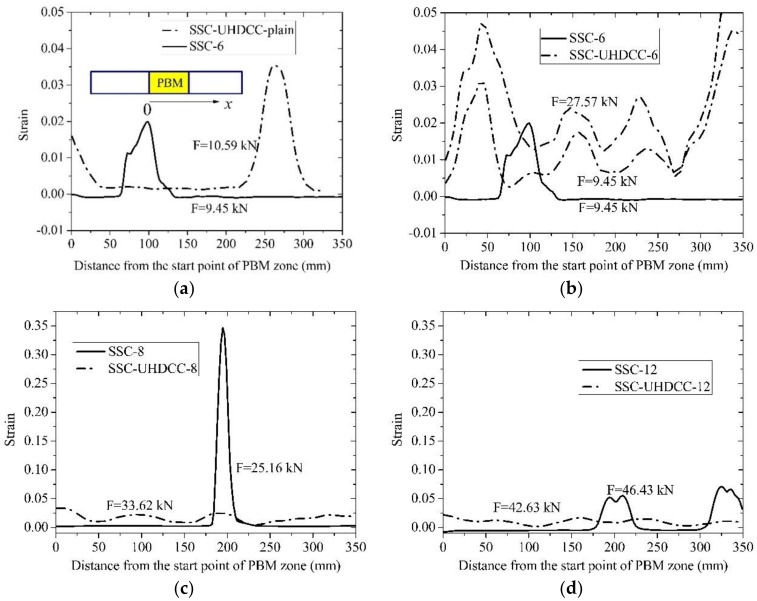
Strain distributions in x direction: (**a**) SSC-UHDCC-plain vs. SSC-6; (**b**) SSC-6 vs. SSC-UHDCC-6; (**c**) SSC-8 vs. SSC-UHDCC-8; and (**d**) SSC-12 vs. SSC-UHDCC-12.

The strain of SCC and UHDCC was extracted from the results of DIC measurement. Figure 17 compares the strain distribution at the level of longitudinal bars of various beams at their ultimate limit state. The area of interest (i.e., the PBM zone of the beam) and the strain direction are illustrated in Figure 17a. The experimental analysis showed the strain distributions in the UHDCC layers had more peaks as evenly distributed cracks in the UHDCC. With the interaction with the FRP bars embedded in the UHDCC layer, the fluctuation was further smoothed, especially with a higher reinforcement ratio. The smooth strain distribution in the concrete reflects the extension in FRP bars fully generated in the PBM zone. Figure 17b compares the strain distributions for two beams at the same moment. The average strain was 0.00129 for SSC-6 and 0.0146 for SSC-UHDCC-6. As the cracked UHDCC layer can still contribute tension load, the larger strain value in the UHDCC layer could alleviate the tension in the FRP bars at the same load capacity. Therefore, this could prolong the function of FRP bars and enhance the flexural capacity.

**Figure 18:**
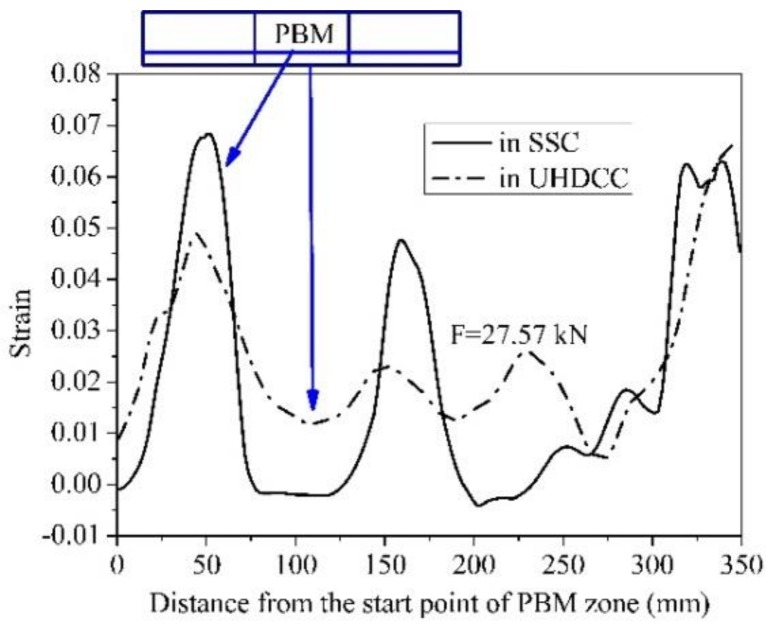
Strain distributions in x direction (in SSC-UHDCC-6).

For further demonstration, Figure 18 illustrates the strain in SSC near the interface and in UHDCC at the location of BFRP bar at a same moment. It is of interest that the local maximum values of SSC strain were larger and the local minimal values of SCC strain were smaller than those of UHDCC at the same x coordinate. This demonstrates the estimate in Figure 16. Based on the strain distribution in Figure 18, the average SCC strain near the interface was calculated to be 0.020, whereas the UHDCC strain was 0.024. The magnitude of strain in UHDCC, which is closer to the tensile side of beam, should be larger than in SCC. We found that the strain in the SSC tensile section in SSC-UHDCC-6 was much larger than in the SSC-6 beam, which further proves that introducing the UHDCC layer can effectively improve the deflection.

To some extent, the strain distributions of SSC and UHDCC in Figure 17 and Figure 18 reflect the strain in the BFRP bar. Due to the brittleness of NC, the tension in the cracked cross-sections of the SSC beam was totally provided by BFRP bars, whereas the tension between adjacent cracks was provided by both BFRP bars and concrete. Therefore, single and large strain fluctuations were observed in the PBM zone of SSC beams in the ultimate state. In contrast, UHDCC can smear one big crack into multiple fine cracks and maintain its tension contribution during cracking; therefore, strain fluctuations are effectively smoothed. 

In summary, for the composite system proposed in this article, the UHDCC layer can act as longitudinal reinforcement. The flexural capacity (both serviceability limits and ultimate state) of the structural system is enhanced due to the tension contribution of UHDCC, and the deformability is improved due to the effective control of crack width by UHDCC, which also prolongs FRP bars’ life before rupture.

## 7. Conclusions

This paper proposes a novel composite structural member composed of sea water and sea sand concrete, UHDCC, and a BFRP bar. UHDCC, a kind of ECC with large rupture strain, was designed to replace the SSC tensile cover to improve the cracking control and bond performance, and the flexural performance in BFRP bar-reinforced SSC beams. The test results demonstrated that the performance, including characteristic load capacity and deformability, crack pattern, and stressing in BFRP bars, could all be improved by introducing UHDCC to the tensile zone. The following conclusions were drawn from this investigation:

(1) With assistance from the DIC sensing method, we discovered that, due to the supremely high rupture strain of UHDCC, the new composite beam can produce multiple and fine cracks in the tensile zone until failure. Its flexural crack width decreases in the concrete section and crack distribution is improved accordingly. Thus, UHDCC limits the extension of cracks and improves the deformability. 

(2) The cracking load, service load, ultimate load, and corresponding deflection of UHDCC-SSC composite beams are higher than SSC beams with the same reinforcement ratio. The enhancements in cracking load, service load, and deformability were significant, without changing beam dimension or the reinforcement ratio. This demonstrates the contribution of the UHDCC layer. 

(3) The multi-cracking characteristic of UHDCC helps to smooth the strain fluctuation in the FRP bar, thus improving the tension efficiency of FRP bars in the case of the beam experiencing flexural failure mechanism. 

To summarize, the new composite beam has resolved the issue of efficient use of FRP bars as tensile reinforcements and the feasible use of FRP bars as sensors. This is largely due to the change in the cracking characteristics and bond performance. Further study is needed on the cracking mechanism and the association with the flexural deformability of FRP bar-reinforced (with optic fibers) concrete beams.

## Figures and Tables

**Table 1:** Mix proportion of sea sand and seawater concrete (SSC) on site.

Cement (PO 42.5) (kg/m^3^)	Coarse Aggregate (kg/m^3^)	Sea Sand (kg/m^3^)	Water (kg/m^3^)	Sea Salt (kg/m^3^)	High-range Water-Reduction (HRWR) (g)
410.00	982.00	803.00	205.00	6.34	0.41

**Table 2:** Mix proportion of ultra-high ductile cementitious composite (UHDCC).

Cement (kg/m^3^)	Fly Ash (kg/m^3^)	Sea Sand (kg/m^3^)	Water (kg/m^3^)	Sea Salt (kg/m^3^)	Polyethylene (PE) Fiber (kg/m^3^)	HRWR (kg/m^3^)
593.00	711.60	474.40	300.00	9.30	19.10	5.00

**Table 3:** Properties of polyethylene (PE) fiber as provided by manufacturer.

Fiber Type	Diameter (μm)	Fiber Aspect Ratio	Tensile Strength (GPa)	Elastic Modulus (GPa)	Rupture Elongation (%)	Density (g/cm^3^)
Polyethylene	24	720	2.9	116	2.42	0.97

**Table 4:** Material properties of BFRP bars.

Diameter of BFRP Bar (mm)	Modulus of Elasticity (GPa)	Peak Tensile Strength (MPa)	Tensile Strain Capacity (%)
6	54.35	1369.62	2.52
8	47.84	1239.06	2.59
12	46.39	937.08	2.02

**Table 5:** Properties of test specimens.

Specimen ID	Diameter of FRP Tensile Bar (mm)	UHDCC Layer (mm)	Longitudinal Reinforcement Ratio (%)
SSC-6	6	0	0.17
SSC-8	8	0	0.30
SSC-12	12	0	0.69
SSC-UHDCC-6	6	60	0.17
SSC-UHDCC-8	8	60	0.30
SSC-UHDCC-12	12	60	0.69
SSC-UHDCC-plain	/	60	/

Note: “SSC” means sea sand and seawater concrete beam; “SSC-UHDCC” means sea sand and seawater beam with UHDCC layer; the number after the capital letters represents the diameter of the longitudinal BFRP bars in the beam; “plain” means there is no longitudinal BFRP bar in the beam.

**Table 6:** Service load using various criteria (kN).

Specimen ID	Service Load Under Different Criteria					*P*_s_ (Floor Level)	*P*_s_/*P*_m_	*P*_s_ (Roof Level)	*P*_s_/*P*_m_
	Δ = *L*/360 (Floor Level)	Δ = *L*/180 (Roof Level)	*ε*_s_	*w*_m_	*P*_m_/1.5				
SSC-UHDCC-plain	7.64	10.51	/	2.53	8.33	2.53	20.26%	2.53	20.26%
SSC-6	7.94	9.16	4.22	2.50	7.46	2.50	22.32%	2.50	22.32%
SSC-UHDCC-6	14.58	17.01	9.23	9.52	20.95	9.52	29.36%	9.52	29.36%
SSC-8	8.27	11.92	1.90	3.63	18.39	3.63	13.16%	3.63	13.16%
SSC-UHDCC-8	14.23	18.49	9.48	15.68	26.36	14.23	35.98%	15.68	39.65%
SSC-12	15.18	22.78	9.07	17.28	31.37	15.18	32.26%	17.28	36.73%
SSC-UHDCC-12	15.51	**23.62**	12.74	36.52	33.11	15.51	31.23%	23.62	47.56%

Note: Δ is the mid-span deflection; *ε*_s_ is the service strain in BFRP bar, set to 0.002; *w*_m_ is the maximum crack width, set to 0.5 mm in SSC; and *P*_m_ and *P*_s_ are the maximum load and service load, respectively.

**Table 7:** Mechanical properties of tested beam.

Specimen ID	Cracking Load (kN)	Service Load (kN)	Ultimate Load (kN)	Deflection at Ultimate Load (mm)
SSC-UHDCC-plain	9.00	2.53	10.59	10.15
SSC-6	2.50	2.50	9.45	10.81
SSC-UHDCC-6	9.00 (260%)	9.52 (281%)	27.57 (192%)	58.07 (437%)
SSC-8	3.60	3.63	25.16	32.69
SSC-UHDCC-8	9.00 (150%)	15.68 (332%)	33.62 (34%)	47.32 (45%)
SSC-12	4.80	17.28	46.43	35.01
SSC-UHDCC-12	12.60 (162.5%)	36.52 (111%)	42.63 (-8%)	47.50 (36%)

Note: The number in parentheses refers to the enhancement ratio of SCC-UHDCC-* specimen to its counterpart SCC-* specimen.

**Table 8:** Crack width and number.

Spec. ID	*w*_5_ (mm)	*w*_8_ (mm)	*w*_10_ (mm)	Crack Number in SSC (mm)
SSC-6	/	/	1.89	3
SSC-UHDCC-6	0	0.37	0.85	10
SSC-8	1.46	1.48	1.78	4
SSC-UHDCC-8	0	0.16	0.26	8
SSC-12	0.37	0.39	0.44	5
SSC-UHDCC-12	0	0	0	9
SSC-UHDCC-plain	0	0.05	5.36	2

Note: *w*_5_, *w*_8_, and *w*_10_ are crack widths in SSC under loads of 5, 8, and 10 kN.
